# Mechanism of Interaction between hsa_circ_0002854 and MAPK1 Protein in PM_2.5_-Induced Apoptosis of Human Bronchial Epithelial Cells

**DOI:** 10.3390/toxics11110906

**Published:** 2023-11-06

**Authors:** Jinchang Hong, Yi Tan, Yuyu Wang, Hongjie Wang, Caixia Li, Wenjia Jin, Yi Wu, Dechun Ni, Xiaowu Peng

**Affiliations:** 1 State Environmental Protection Key Laboratory of Environmental Pollution Health Risk Assessment, South China Institute of Environmental Sciences, Ministry of Ecology and Environment, Guangzhou 510535, China; inkhong123@163.com (J.H.); tanyi@scies.org (Y.T.); wangyuyu@scies.org (Y.W.);; 2 School of Public Health, China Medical University, Shenyang 110122, China

**Keywords:** atmospheric fine particles, human bronchial epithelial cells, apoptosis, MAPK1

## Abstract

Fine particulate matter (PM_2_._5_) pollution increases the risk of respiratory diseases and death, and apoptosis is an important factor in the occurrence of respiratory diseases caused by PM_2.5_ exposure. In addition, circular RNAs (circRNAs) can interact with proteins and widely participate in physiological and pathological processes in the body. The aim of this study was to investigate the mechanism of circRNA and protein interaction on PM_2.5_-induced apoptosis of human bronchial epithelial cells (16HBE) in vitro. In this study, we exposed human bronchial epithelial cells to a PM_2.5_ suspension with different concentration gradients for 24 h. The results showed that apoptosis of 16HBE cells after PM_2.5_ treatment was accompanied by cell proliferation. After exposure of PM_2.5_ to 16HBE cells, circRNAs related to apoptosis were abnormally expressed. We further found that the expression of hsa_circ_0002854 increased with the increase in exposure concentration. Functional analysis showed that knocking down the expression of hsa_circ_0002854 could inhibit apoptosis induced by PM_2.5_ exposure. We then found that hsa_circ_0002854 could interact with MAPK1 protein and inhibit MAPK1 phosphorylation, thus promoting apoptosis. Our results suggest that hsa_circ_0002854 can promote 16HBE apoptosis due to PM_2.5_ exposure, which may provide a gene therapy target and scientific basis for PM_2.5_-induced respiratory diseases.

## 1. Introduction

In the past few decades, air pollution has become a major factor affecting human health. More than 90% of the world’s population lives in places where air quality levels are well below World Health Organization (WHO) standards. It is estimated that 40,000 people worldwide die each year from lung cancer, heart disease, stroke, and acute and chronic airway diseases due to ambient air pollution [1]. The pollutants that have the greatest impact on human health include particulate matter (PM), sulfur dioxide, ozone, and nitrogen dioxide. The harmful effects of PM on human health have become a major concern for governments and health organizations around the world. PM consists of solid and liquid droplets suspended in the atmosphere. PM are generally divided into three categories based on their aerodynamic diameters: coarse particles (PM_2_._5–10_) with a diameter of 2.5–10 μm, fine particles (PM_2.5_) with a diameter equal to or less than 2.5 μm, and ultrafine particles with a diameter of less than 0.1 μm. Different sources and chemical compositions of PM may lead to different health effects. PM_2.5_ has a relatively small particle size but a large surface area, which makes it easier to absorb various toxic substances. In 2015, the global burden of disease ranked PM_2.5_ as the fifth risk factor for death [2]. PM_2.5_ can enter the lungs through respiration, be deposited in the terminal bronchioles and alveoli, and even be transported to other tissues and organs through the circulatory system, causing multiple organ damage [3]. Studies [4,5] have found that PM_2.5_ exposure is significantly associated with respiratory hospitalization rates and mortality. Other studies [6] have shown that PM_2.5_ can cause apoptosis of human bronchial epithelial cells. Therefore, how does PM_2.5_ affect the respiratory system after entering the human body, and what impact does it have on the cells of the respiratory system? What is the mechanism of molecular action? At present, there are few reports from the epigenetic level, and further exploration is still needed. Therefore, it is of great significance to reveal the negative effects of PM_2.5_ on the human body, which can provide a scientific basis for disease diagnosis and clinical treatment.

PM_2.5_ is associated with a wide range of adverse health effects, including nervous system (e.g., dementia), metabolic (e.g., diabetes), allergies (e.g., rhinitis), kidney, inflammatory, and autoimmune disorders, and lower respiratory tract infections [7]. However, from a public health perspective, the impact on cardiopulmonary disease is critical [8]. Both short- and long-term exposure can lead to the exacerbation of asthma and chronic obstructive pulmonary disease (COPD), as well as worsening lung function and increasing the incidence of COPD [7]. “Air pollution: a major threat to lung health”, the Lancet wrote. It was once thought that chronic obstructive pulmonary disease (COPD) was the main consequence of smoking, but despite tobacco control measures, it remains a worldwide burden, and environmental particulate matter is now recognized as a major risk factor for COPD [9]. There is growing evidence that short- or long-term exposure to PM_2.5_ significantly increases morbidity and mortality from lung diseases, including pulmonary fibrosis, pneumonia, asthma, chronic obstructive pulmonary disease, and even lung cancer [2,10].

Several studies have shown that PM_2.5_ can promote cell apoptosis. Zhang et al. indicated that PM_2.5_ could activate inflammatory axis of COX-2/PGES/PGE2 in vascular endothelial cells to promote cell apoptosis and inflammatory response [11]. Wang et al. [12] verified at the cellular level that PM_2.5_ can induce autophagy and apoptosis in human endothelial cells through endoplasmic reticulum stress. Other studies have shown [6] that PM_2.5_ can trigger apoptosis of lung epithelial cells through the mitochondrial apoptosis pathway mediated by the mitochondrial fission axis of ROS-DRP1. Many studies [13,14,15,16,17] have shown that apoptosis can cause respiratory diseases such as chronic obstructive pulmonary disease, asthma, idiopathic pulmonary fibrosis (IPF), acute respiratory distress syndrome (ARDS), and pulmonary hypertension (PAH). 

Circular RNAs (circRNAs) are covalently closed single-stranded transcripts composed of many RNA species. One type of circRNA, discovered more than 20 years ago, is produced by reverse splicing of precursor mRNA (pre-mRNA) in higher eukaryotes. However, it is thought that these circRNAs are by-products of abnormal splicing within cells and have little functional potential. RNA sequencing (RNA-SEQ) showed that circRNA expression was widely present in the detected cells and tissues, and more than 10% of the expressed genes could produce circRNA. According to current reports [18,19] on the function of circRNAs, circRNAs can adsorb miRNAs, regulate transcription, regulate parental genes, serve as protein complex scaffolds, and facilitate circRNA–protein interactions. Studies [20] have found that circRNA can interact with proteins to exert its regulatory function. According to the current research, circRNA can interact with proteins in five ways, namely, changing the interaction between proteins, binding or sequestering proteins, recruiting proteins to chromatin, forming circRNA-protein-mRNA ternary complexes, and transporting or redistributing proteins.

CircRNA is widely involved in regulating physiological and pathological processes and diseases by interacting with proteins. As a widely existing non-coding RNA with important regulatory functions in living organisms, circRNA must be associated with other important biological macromolecules, and circRNA must also be associated with proteins related to apoptosis. Therefore, we propose a scientific hypothesis that circRNA and protein interaction play an important role in the mechanism of PM_2.5_-induced apoptosis in human bronchial epithelial cells. 

In this study, the mechanism of circRNA and protein interaction in PM_2.5_-induced apoptosis of human bronchial epithelial cells was investigated in vitro, aiming to provide gene therapy targets and a scientific basis for PM_2.5_-induced apoptosis of human bronchial epithelial cell-induced respiratory diseases at the circRNA regulatory level.

## 2. Materials and Methods

### 2.1. Cell and Cell Culture

Human bronchial epithelial cells (16HBE) were purchased from the US ATCC cell bank. 16HBE cells were grown in KM complete medium (Sciencell, Carlsbad, CA, USA) consisting of 1% penicillin/streptomycin, 1% KGS, and 98% KM basal medium in a 5% CO_2_ atmosphere at 37 °C.

### 2.2. Collection and Treatment of Particulate Matter

The TH-1000Ⅱ (Wuhan Tianhong, Wuhan, China) large flow particle sampler was used to collect atmospheric particles. The sampling time is from February to March 2021, and the sampling place is on the roof of the office building of Ruihe Road, Huangpu District, Guangzhou City, Guangdong Province, close to the main road of the transportation hub. The sample collection flow rate was 1.05 m^3^/min, and the filter membrane was replaced after continuous collection for 72 h. The quartz filter membrane absorbed with particles was cut into a size of about 1 cm × 1 cm and put into a large beaker. Ultra-pure water was added and stirred with a glass rod. After ultrasonic oscillation on an ultrasonic oscillator for 1 h, the suspension of particles was filtered through six layers of gauze to prepare PM_2.5_ suspension. The suspension was divided into conical bottles, covered with tin foil (poked with several small holes), frozen in an ultra-low temperature refrigerator at −80 °C overnight, and then dried by a vacuum freeze dryer for 3 days. After removing the quartz filter fiber, the dried particles were weighed, and a high concentration of 10 mg/mL was prepared with PBS (Biosharp, Guangzhou, China), then autoclaved and stored in a refrigerator at 4 °C for later use. The remaining particles were labeled, transferred to a centrifuge tube, and stored in a −20 °C refrigerator.

### 2.3. Cell Counting Kit-8 Assay

Cell Counting Kit-8 (CCK-8) (Beyotime, Shanghai, China) was used to measure cell viability. Human bronchial epithelial cells were cultured in 24-well plates and treated with PM_2.5_ for 24 h, with 3 biological replicates per group. The number of cells per well was 2.5 × 10^4^. An aliquot of 100 μL of CCK-8 reagent was added to each well and reacted at 37 °C for 2 h. The optical density was measured at 450 nm with a microplate reader (BioTek Synergy HT, Winooski, VT, USA). Cell viability was measured according to the instructions.

### 2.4. Flow Cytometry Experiment

Cells at a density of 2.5 × 10^5^ 16HBE were cultured in 24-well plates. All 16HBE cells in the wells were collected 24 h after exposure. Cells were stained using the Annexin V-FITC/PI kit (Beyotime, China). Analysis was performed by Guava^®^ easyCyte™ flow cytometry (Luminex, Austin, TX, USA). Total apoptosis rate = early apoptosis rate (Annexin V-FITC(+), PI(−)) + late apoptosis rate (Annexin V-FITC(+), PI(+)).

### 2.5. RT-qPCR Analysis

Cells were lysed by RNAex Pro reagent (Accurate Biology, Guangzhou, China), and total RNA was isolated according to RNAex Pro reagent’s instructions. Total RNA was reverse transcribed using an Evo M-MLV RT Kit with gDNA Clean for qPCR II (Accurate Biology, China). The RNA sample was divided into two equal parts for qRT-PCR. One part was incubated with Ribonuclease R (RNase R) (Geneseed, Guangzhou, China) at 37 °C for 3 min to digest linear RNA and enrich circRNA. The other part was not treated with RNase R to detect mRNA. qRT-PCR was conducted using SYBR^®^ Green Pro Tap HS (Accurate Biology, China) and detected by Step One Plus (Applied Biosystems, Carlsbad, CA, USA). Relative expression levels of the genes were calculated according to the 2^−ΔΔCt^ method, where Ct represents cycle threshold number, ΔCt is the difference in Ct between the target and reference genes, and ΔΔCt represents the relative change between the treatment and blank groups. ACTB (β-actin) was used as an internal reference gene to correct relative gene expression. The primers are exhibited in Table 1.

### 2.6. RNase R Digestive Experiment

The experiment was divided into an RNase R (+) group (treated with RNase R) and an RNase R (−) group (treated without RNase R). Total RNA was treated according to the above groups, and qRT-PCR was performed. The RNase R experiment was conducted in accordance with the RNase R specification (Geneseed, Guangzhou, China).

### 2.7. Nucleocytoplasmic Separation Experiment

About 10^7^ 16HBE cells were collected. Cytoplasmic and nuclear RNA were extracted according to the procedures of the cytoplasmic and nuclear RNA purification kits (Invitrogen, Carlsbad, CA, USA). Then, cytoplasmic RNA and nuclear RNA were converted into cDNA for qRT-PCR analysis. In this study, β-actin and U6snRNA were used as controls.

### 2.8. Fluorescence In Situ Hybridization Experiment (FISH)

16HBE cells were cultured in confocal petri dishes. When the cell density exceeded 50%, the cells were treated according to the instructions of the FISH kit (GenePharma, Suzhou, China) and incubated with the probe (Geneseed, Guangzhou, China). Finally, the petri dishes were observed and photographed with an IX71 microscope (Olympus, Shinjuku, TKY, Japan). The FISH probe was designed by Guangzhou Geneseed Biological Technology Co., Ltd., Guangzhou, China.

hsa_circ_0002854 FISH probe sequence:

5′CY3-GCCACTCGTACCTGTATAACTAG 3′CY3

### 2.9. Small Interfering RNA (siRNA) Transfection

For gene knockdown, small interfering RNA (siRNA) of has_circ_0002854 was transferred into 16HBE cells via the Lipofectamine^®^ 3000 Reagent kit (Thermo Scientific, Waltham, MA, USA). The final siRNA transfection concentration was 60 nM, and the transfection time was set at about 6 h.

### 2.10. RNA Pull-Down Experiment

The RNA pull-down kit was purchased from BersinBio Co., Ltd. (Guangzhou, China). Perform the RNA pull-down according to kit instructions. The simple steps are as follows: At first, the probe hsa_circ_0002854 labeled with 2 μg biotin was formed into a secondary structure and adsorbed on streptavidin magnetic beads to form a probe-magnetic bead complex. The whole protein was then extracted from about 2 × 10^7^ 16HBE cells, and the nucleic acid was removed from the sample. The probe magnetic bead complex and the cell extract were mixed together and incubated at room temperature for 3 h. Finally, RNA-binding proteins (RNPs) were collected and identified using a silver staining kit (Sangon, Shanghai, China). The mass spectrometry experiment was completed by Guangzhou Ruqi Biotechnology Co., Ltd., Guangzhou, China. RNA pull-down experiments were performed only once, and we processed the enrichment values of 0 μg/mL and 200 μg/mL protein when analyzing the mass spectrometry data. In brief, the protein enrichment values of the RPD group in the 0 μg/mL and 200 μg/mL groups were compared with the protein enrichment values of the NC group, and the actual enriched protein values of hsa_circ_0002854 in the 0 μg/mL and 200 μg/mL groups were obtained, respectively. Then, the actual enriched protein value in the 200 μg/mL group was compared with the actual enriched protein value in the 0 μg/mL group to obtain the actual enriched protein value after PM_2.5_ treatment. Finally, the data were uploaded to the *Metascape* (https://metascape.org/ (accessed on 25 December 2022)) website for bioinformatics analysis to screen out the most likely interacting proteins. The probe sequence is shown in Table 2.

### 2.11. Protein Extraction and Western Blot Analysis

The cells were lysed on ice with RIPA buffer containing protease inhibitor and phosphatase inhibitor (MCE, Monmouth Junction, NJ, USA) for 30 min, followed by centrifugation of the lysate at 14,000× *g* for 15 min with total protein supernatant for analysis. Thermo Fisher BCA protein detection kit was used to detect the protein concentration of the supernatant. The protein sample was mixed with 6x loading buffer and denatured at 100 °C for 5 min. Preparation of 10% separated polyacrylamide gel. Each gel hole was filled with 40 μg of total protein samples for electrophoresis. The isolated proteins are transferred to the membrane. These membranes were blocked with 5% bovine serum albumin and incubated for 1 h. These membranes are reacted with the following primary antibodies at 4 °C for 12 h:p-ERK1 (1:300, Wanleibio, Shenyang, China, catalog number: WLP1512), MAPK1 (1:1000, Solarbio, Beijing, China, catalog number: K000191M), and beta-tubulin (1:5000, Solarbio, China, catalog number: K200059M). The membranes were shaken with Tris buffered saline Tween-20 (TBS-T) buffer for 15 min and reacted with IRDye^®^800CW goat anti-rabbit secondary antibody (1:10,000, LI-COR, Lincoln, NE, USA) or goat anti-mouse secondary antibody (1:10,000, AmyJet, Guangzhou, China) at room temperature for 1 h. Then Odyssey (LI-COR) was used to scan and photograph these films, and imageJ software was used to analyze the gray values of the band. Beta-tubulin is an internal reference protein.

### 2.12. Statistical Analysis

Experimental data are expressed as mean ± SEM, and experiments were repeated at least three times except for RNA pull-down (which was performed once). SPSS 27.0 (IBM, Armonk, NYC, USA) was used for statistical analysis of the data, and GraphPad Prism 9.0 (GraphPad Software, San Diego, CA, USA) was used to plot the experimental results. The LSD-*t* test was used for comparison between two groups, and a one-way ANOVA was used for comparison between multiple groups. When *p* < 0.05, the difference was statistically significant.

## 3. Result

### 3.1. Survival Rate of 16HBE Cells after 24 h of Exposure to PM_2.5_

**Figure 1:**
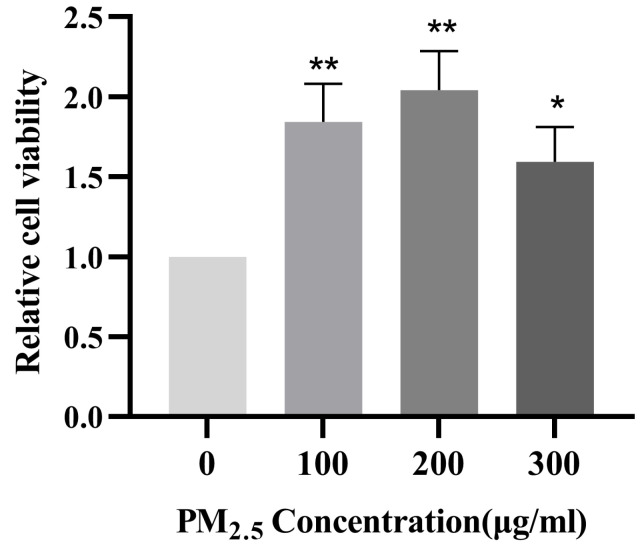
Effects of PM_2.5_ on the viability of 16HBE cells. Compared with the control group, * *p* < 0.05, ** *p* < 0.01.

16HBE cells were treated with PM_2.5_ concentrations of 0, 100, 200, and 300 μg/mL, respectively, for 24 h. As shown in Figure 1, after 16HBE cells were exposed to different concentrations of PM_2.5_, the results showed that the cell survival rate at 100, 200, and 300 μg/mL concentrations was higher than that of the control group. When the concentration of PM_2.5_ was 100, 200, and 300 μg/mL, the difference was statistically significant.

### 3.2. Apoptosis Rate of 16HBE Cells after 24 h of Exposure to PM_2.5_

**Figure 2:**
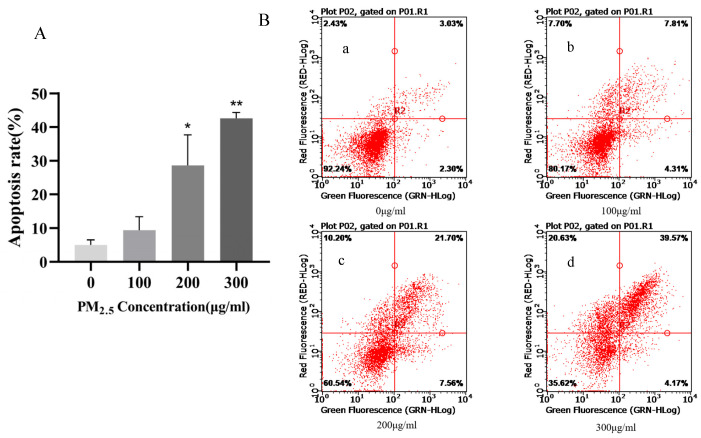
Effects of PM_2.5_ on the apoptosis rate of 16HBE cells. (**A**) is a histogram showing the increase in total apoptosis rate (sum of early and late apoptosis rates) with the increase in PM_2.5_ exposure concentration. In (**B**), (**a**–**d**) is a scatter plot showing the increase in apoptosis rate with the increase in PM_2.5_ exposure concentration. The lower left quadrant (Annexin V−/PI−) represents normal cells; the Annexin V+/PI− and Annexin V+/PI+ quadrants represent early and late apoptotic cells, respectively. Compared with the control group, * *p* < 0.05, ** *p* < 0.01.

16HBE cells were exposed to PM_2.5_ concentrations of 0, 100, 200, and 300 μg/mL for 24 h, and the cells were collected and treated with the Annexin V-FITC/PI apoptosis assay kit, and then the apoptosis rate was detected by flow cytometry. As shown in Figure 2A,B, compared with the control group, the apoptosis rate increased with the increase in PM_2.5_ exposure concentration. With the increase in PM_2.5_ exposure concentration, the apoptosis rate changed significantly and showed a significant dose-response relationship with the increase in exposure concentration. Compared with the control group, the difference was statistically significant except for the concentration of 100 μg/mL. The results showed that PM_2.5_ could increase the apoptosis rate. According to the experimental results and cell morphology observed under the microscope, 0, 200, and 300 μg/mL PM_2.5_ concentrations were selected as the exposure conditions for subsequent experiments.

### 3.3. qRT-PCR-Validated circRNAs Associated with Apoptosis Pathways

**Figure 3:**
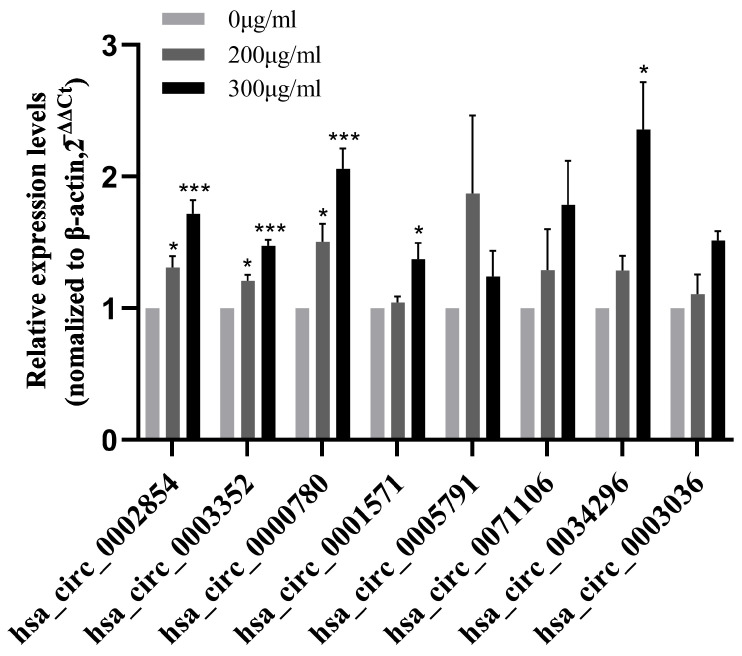
CircRNA expression level in 16HBE cells exposed to PM_2.5_ for 24 h. Compared with the control group, * *p* < 0.05, *** *p* < 0.001.

According to the previous high-throughput sequencing results of our research group, eight circRNAs with differential expression related to apoptosis pathways were screened out. In order to verify the accuracy of the results of RNA-seq, qRT-PCR verification was performed on these eight circRNAs (Figure 3).

### 3.4. RNase R Digestive Test

**Figure 4:**
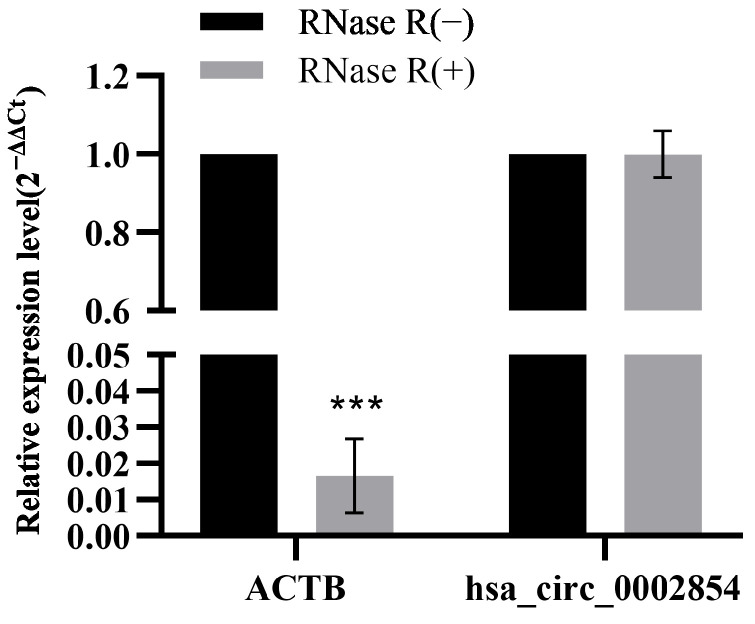
RNase R digestion experiment. Compared with the control group, *** *p* < 0.001.

The total RNA of 16HBE cells was treated with RNase R to detect the expression of β-actin (ACTB) and hsa_circ_0002854. As can be seen from the experimental results, the expression level of ACTB was significantly decreased, with statistical significance (*p* < 0.001), while the expression level of hsa_circ_0002854 was almost unchanged (Figure 4). The experimental results show that hsa_circ_0002854 is resistant to RNase R.

### 3.5. Nucleocytoplasmic Separation Experiment

**Figure 5:**
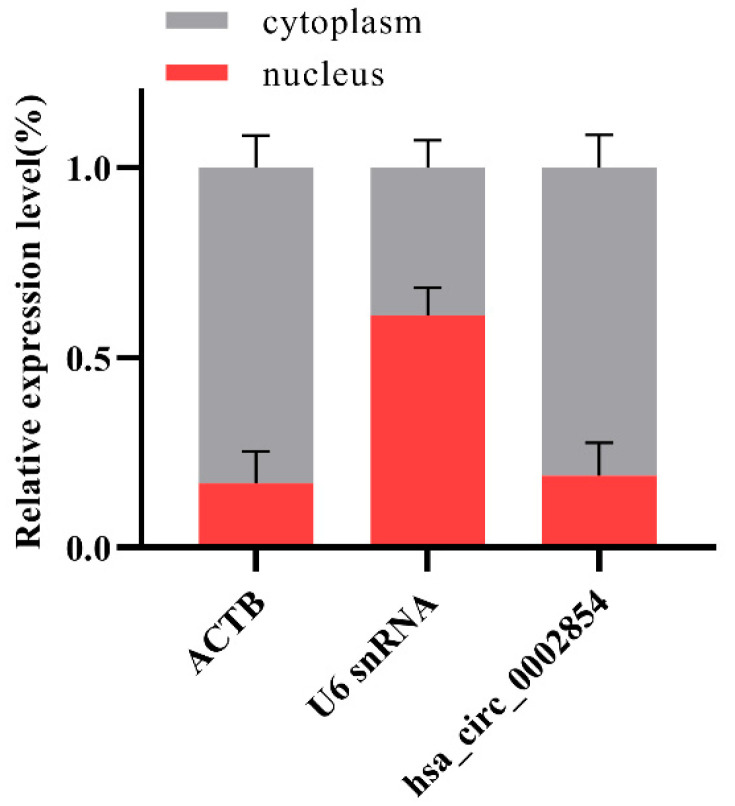
Nucleocytoplasmic separation experiment.

RNA after nucleocytoplasmic separation of 16HBE cells was detected by reverse transcription and qRT-PCR, as shown in Figure 5. The experimental results showed that the internal reference ACTB and U6 snRNA (U6 small nuclear RNA) were mainly expressed in the cytoplasm and nucleus, respectively, suggesting that the nuclear cytoplasmic separation experiment was successful and hsa_circ_0002854 was mainly expressed in the cytoplasm.

### 3.6. Fluorescence In Situ Hybridization Experiment

**Figure 6:**
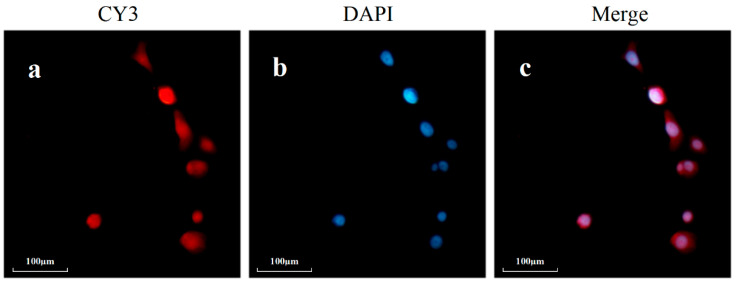
Results of the fluorescence in situ hybridization experiment. (**a**) is hsa_circ_0002854 marked with CY3; (**b**) is the cells stained by DAPI; (**c**) is the overlapping diagram of cells after staining.

To further verify the distribution of hsa_circ_0002854 in cells, we localized hsa_circ_0002854 through a fluorescence in situ hybridization experiment and found that Cy3-labeled hsa_circ_0002854 was distributed in both the nucleus and cytoplasm, as shown in Figure 6.

### 3.7. Interference Efficiency of siRNA

**Figure 7:**
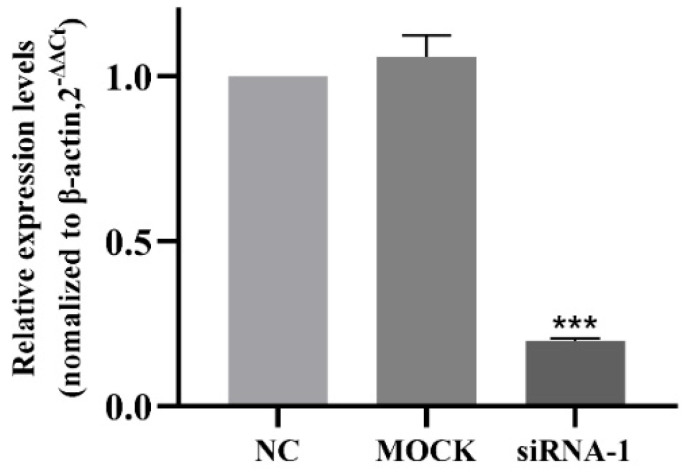
Expression level of hsa_circ_0002854 after siRNA interference. Compared with the control group, *** *p* < 0.001.

After interfering with hsa_circ_0002854 in 16HBE cells, the interference efficiency of siRNA-1 was detected by qRT-PCR. According to the experimental results, the relative expression levels of the NC group and MOCK group were close, and the difference was not statistically significant. Compared with the NC group, the expression level of hsa_circ_0002854 in the siRNA treatment group was significantly decreased, and the interference efficiency was over 80%. The difference was statistically significant (*p* < 0.001). The results showed that siRNA-1 could effectively interfere with the expression level of hsa_circ_0002854, and the sequence could be used for subsequent interference experiments. The results are shown in Figure 7.

### 3.8. hsa_circ_0002854 Functional Verification Experiment

**Figure 8:**
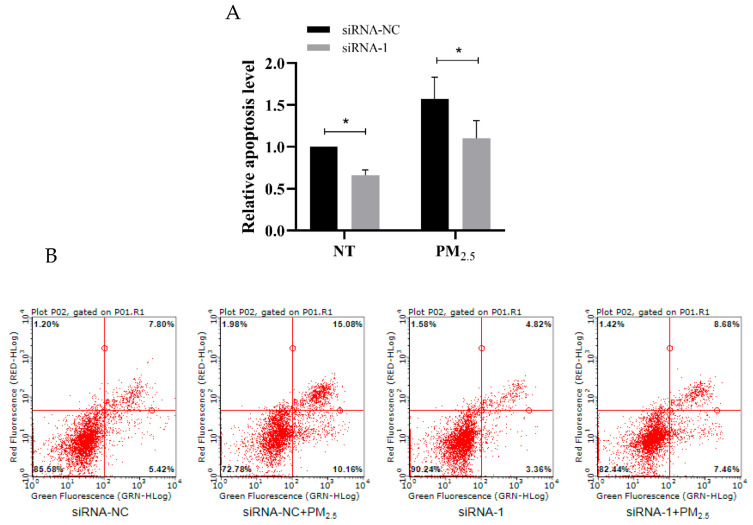
Apoptosis level of 16HBE cells after siRNA interference. (**A**) shows the relative apoptotic level (sum of early apoptotic cells and late apoptotic cells) of 16HBE cells exposed to PM_2.5_ after interference and non-interference with hsa_circ_0002854. (**B**) is a flow scatter plot with Annexin V−/PI− in the lower left quadrant representing normal cells. Annexin V+/PI− in the lower right quadrant and Annexin V+/PI+ in the upper right quadrant represent early apoptotic cells and late apoptotic cells. Compared with the control group, * *p* < 0.05.

Flow cytometry results showed that after interfering with the expression of hsa_circ_0002854 in 16HBE cells, the apoptosis rate of treated and non-treated groups was significantly decreased compared with the control group, with statistical significance (*p* < 0.05), as shown in Figure 8A,B. These results indicate that the apoptosis of 16HBE cells can be inhibited by interfering with the expression of hsa_circ_0002854 in vitro.

### 3.9. Study on the Mechanism of hsa_circ_0002854

**Figure 9:**
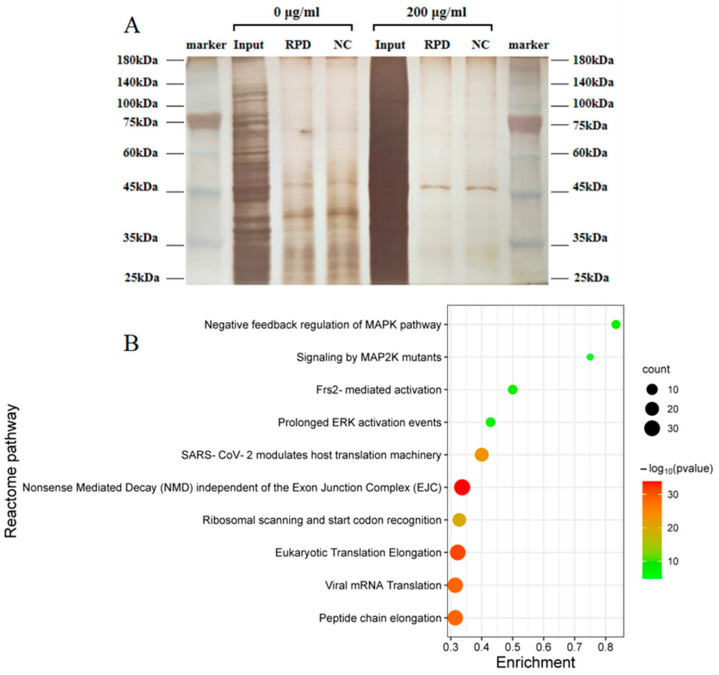
RNA pull-down results. (**A**) is the silver staining diagram of protein after RNA pull-down; (**B**) is the enrichment diagram of the *Reactome* pathway after protein profiling.

Using linear hsa_circ_0002854 DNA and a small segment of splitter DNA as templates, hsa_circ_0002854 with biotin tags and sense and antisense RNA with splitter sequences were transcribed in vitro and then incubated with 16HBE cell lysate, respectively. The pull-down proteins were dyed silver to observe whether there were different bands between the sense chain group and the antisense chain group. The experimental results showed that hsa_circ_0002854 was able to bind more proteins (Figure 9A). After the pulled-down proteins were performed by mass spectrometry, the proteins with high differential expression multiples were screened out and uploaded to *Metascape* (https://metascape.org/ (accessed on 25 December 2022)) for analysis. Based on the enrichment of the *Reactome* pathway, it is speculated that hsa_circ_0002854 may interact with the MAPK signaling pathway (Figure 9B).

### 3.10. Western Blot Assay Verified the Regulatory Effect of hsa_circ_0002854 on MAPK1 Protein

**Figure 10:**
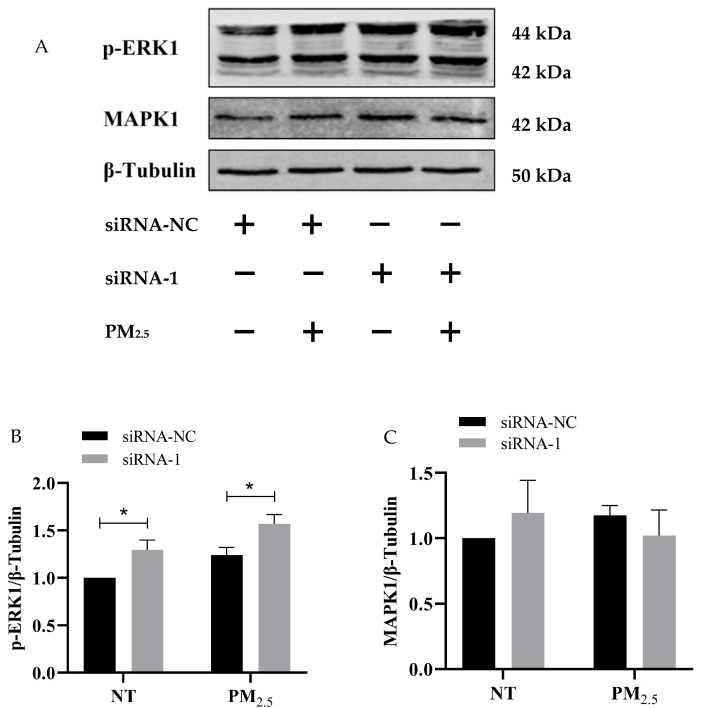
Expression levels of p-ERK and MAPK1 after siRNA interference. (**A**) shows the results of the western blot test. (**B**) shows the histogram of relative expression of the p-ERK1 protein. (**C**) shows the histogram of the relative expression of the MAPK1 protein. Compared with the control, * *p* < 0.05.

Western blot assay (Figure 10A) showed that interference with hsa_circ_0002854 resulted in an increase in the relative expression of p-ERK1 between the exposed group and the non-exposed group (Figure 10B), with statistical significance (*p* < 0.05). However, the difference in MAPK1 protein was not statistically significant (Figure 10C). From the results of this experiment, it can be concluded that interference with hsa_circ_0002854 can promote the expression level of the p-ERK1 protein. In addition, hsa_circ_0002854 can interact with the MAPK1 protein and inhibit its phosphorylation. 

## 4. Discussion

Apoptosis is an important reason for the occurrence and development of respiratory diseases [21]. Studies [22] have shown that PM_2.5_ can induce a variety of cell death modes, including autophagy, necrosis, apoptosis, pyroptosis, and ferroptosis. This study focuses on the mechanism of PM_2.5_-induced apoptosis in 16HBE cells, and the exploration of other mechanisms will be carried out when we have more resources in the future. In the concentration settings of PM_2.5_-treated cells, we referred to some studies [23,24,25]. We found that the PM_2.5_ concentration settings were different, including equal ratio and equal difference, but most of them were below 400 μg/mL. In order to better explore the effect of apoptosis, we set the PM_2.5_ concentration at 0, 100, 200, and 300 μg/mL. Then, 16HBE cells were treated with 0, 100, 200, and 300 μg/mL PM_2.5_ solution in serum-free medium for 24 h, and the cell survival rate was detected by CCK-8 and the apoptosis rate was detected by flow cytometry. The results of the experiment showed a very interesting phenomenon: the cell survival rate of 100, 200, and 300 μg/mL concentrations was higher than 0 μg/mL, but the apoptosis rate increased with the increase in PM_2.5_ concentration, indicating that after the treatment of 16HBE cells with PM_2.5_, cell apoptosis was accompanied by cell proliferation. However, relevant studies have pointed out that this seemingly paradoxical phenomenon does indeed exist. Huang et al. [26] showed that dying tumor cells use the apoptotic process to generate powerful growth-stimulating signals to stimulate the regeneration of tumors receiving radiotherapy. The mechanism is that one of the downstream effector targets regulated by caspase3 is prostaglandin E2 (PGE2), which can effectively stimulate the growth of surviving tumor cells. Apoptosis-stimulated tissue regeneration has also been observed in lower organisms, such as Drosophila melanogaster and the polyp system [27,28,29]. In these cases, it has been proposed that apoptotic cells stimulate the so-called compensatory proliferation of tissue regeneration. Therefore, we speculate that 16HBE cells may also undergo compensatory proliferation of so-called tissue regeneration in this experiment, but we cannot conclude, and further mechanisms remain to be investigated.

The purpose of this study is to explore the apoptosis effect of PM_2.5_ on 16HBE cells after exposure. We found that when the concentration of PM_2.5_ was between 200 and 300 μg/mL, the apoptosis rate was significantly increased compared with the control group, which was statistically significant. Therefore, 16HBE cells were treated with PM_2.5_ concentrations of 0, 200, and 300 μg/mL for 24 h as the exposure concentration for subsequent experiments. In addition, the above results fully proved that PM_2.5_ could increase the apoptosis rate and induce apoptosis in 16HBE cells, and the cell apoptosis model was successfully constructed.

circRNA is a research hotspot in epigenetics at present. Compared with linear RNA, circRNA has better stability and can interact with proteins, adsorb miRNA, encode proteins, etc. [18]. Therefore, in the subsequent experiments, based on the previous high-throughput sequencing results of our research group, we screened out circRNAs related to apoptosis signaling pathways and further studied the mechanism of circRNA in PM_2.5_-induced apoptosis of 16HBE cells. Sequencing results showed that the abnormal expression of circRNAs occurred in 16HBE cells after PM_2.5_ exposure. In order to further study the role of these abnormally expressed circRNAs in apoptosis, we screened eight apoptosis-related circRNAs from the apoptosis pathway, the P53 pathway, the TNF pathway, etc. Firstly, 16HBE cells were exposed to PM_2.5_ at concentrations of 0, 200, and 300 μg/mL for 24 h, and then total RNA was extracted and detected by qRT-PCR. The results showed that the expression levels of hsa_circ_0002854, hsa_circ_0003352, and hsa_circ_0000780 increased with the increase in exposure concentration and were statistically significant, while the expression levels of other circRNAs showed no obvious trend or statistical significance. After screening the above three circRNAs, we have carried out pre-experiments for functional verification. We have only carried out 1–2 experiments in the pre-experiment stage. This part of the data is only for our research reference, which needs to be further studied for publication as a paper. Interference with hsa_circ_0003352 and hsa_circ_0000780 did not show a significant trend in apoptosis rate, while interference with hsa_circ_0002854 resulted in a significant decrease in apoptosis rate. According to the experimental results, we finally selected hsa_circ_0002854 to continue the subsequent functional experiments, and further research on hsa_circ_0003352 and hsa_circ_0000780 will be carried out when we have spare capacity.

In order to further verify whether hsa_circ_0002854 has a regulatory function in the apoptosis process, we first performed a cell nucleoplasmic separation experiment. The purpose of this experiment was to lay the foundation for the later interference experiment because, when circRNA is distributed in the cytoplasm or nucleus, the interference method is not the same [30,31]. The results showed that hsa_circ_0002854 was mainly distributed in the cytoplasm. Next, we successfully used siRNA to interfere with hsa_circ_0002854. At the same time as reducing the expression level of hsa_circ_0002854 in the cells, we treated 16HBE cells with PM_2.5_, and we found that the apoptosis rate of the exposed group and the non-exposed group was significantly reduced, and the results were statistically significant. Based on the experimental results, we concluded that hsa_circ_0002854 had the function of promoting apoptosis.

In this experiment, we hypothesized that hsa_circ_0002854 could interact with proteins. According to relevant studies [32], the linear form of the probe used for circular RNA pull-down can be used, but whether there is a difference in binding protein between the linear form and the circular form of the probe remains to be studied. Then, we carried out an RNA pull-down experiment on hsa_circ_0002854. Although our RNA pull-down experiment was only conducted once, the concentration group we set had the function of comparison and could also present the pull-down results objectively. In the subsequent experiments, we further validated the RNA pull-down results by performing three WB experiments on the predicted proteins. After the RNA pull-down experiment was completed, we performed silver staining and mass spectrometry experiments on the pulled-down proteins. Silver staining results showed that hsa_circ_0002854 could interact with many proteins. After the mass spectrometry results were obtained, we uploaded them to the *Metascape* website for bioinformatics analysis. Finally, the predicted results from the website showed that hsa_circ_0002854 could interact with proteins in the MAPK pathway. Qi et al. suggested that PM_2.5_ could cause apoptosis and cell damage by activating the MAPK/NF-кB/STAT1 pathway in A549 cells [33]. Our study together showed that PM_2.5_ could induce cell apoptosis through the MAPK pathway. In order to verify the experimental results of RNA pull-down, we performed WB experiments on the key proteins of the MAPK pathway, and we finally selected MAPK1 and p-ERK1 for the experiments. MAPK1 protein is the total protein in the downstream stage of the MAPK pathway; its change is relatively stable, so its change is small and not statistically significant. p-ERK1, as a downstream cascade protein in the MAPK pathway, is the phosphorylated form of MAPK1. P-ERK1 can enter the nucleus to initiate transcription and promote cell proliferation [34]. Since circRNA can bind to proteins and affect their transport process, we speculated that hsa_circ_0002854 could interact with MAPK1 protein and inhibit the phosphorylation of MAPK1 protein; in other words, inhibit the entry of p-ERK1 into the nucleus and promote cell proliferation. Thus far, hsa_circ_0002854 has not been reported to be involved in PM_2.5_-induced apoptosis or other diseases.

**Figure 11:**
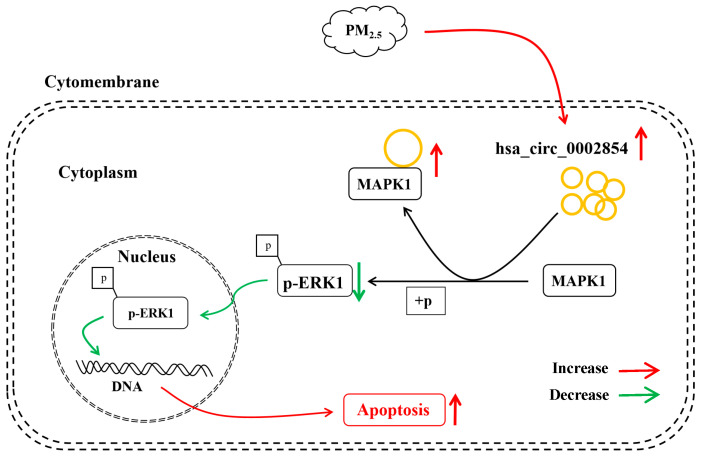
Mechanism diagram of apoptosis induced by PM_2.5_ in 16HBE cells.

This study confirmed that hsa_circ_0002854 could promote the apoptosis of 16HBE cells caused by PM_2.5_ exposure. hsa_circ_0002854 could interact with MAPK1 protein to inhibit the phosphorylation of MAPK1 protein and promote apoptosis. The deeper regulatory mechanism of hsa_circ_0002854 needs to be further studied, and hsa_circ_0002854 may become a new therapeutic target for PM_2.5_-induced respiratory diseases. The mechanism diagram of this experiment is shown in Figure 11.

## 5. Conclusions

Hsa_circ_0002854 plays an important role in PM_2.5_-induced apoptosis of human bronchial epithelial cells and can interact with MAPK1 to inhibit the phosphorylation of MAPK1 and promote cell apoptosis. These findings provide new insights into the treatment of respiratory diseases caused by PM_2.5_.

## Figures and Tables

**Table 1:** circRNA primer sequence list.

Primer	Primer Sequence (5′ to 3′)
ACTB-F	TCTGGCGGCACCACCATGTAC
ACTB-R	CTGCTTGCTGATCCACATCTGCTG
U6_F1	CTCGCTTCGGCAGCACA
U6_R1	AACGCTTCACGAATTTGCGT
hsa_circ_0002854_F	GTCAGCTCAAACCACTAGTTATACAGGTAC
hsa_circ_0002854_R	CAGTCATCGTCATCATCATTTTCGTCC
hsa_circ_0003036_F	GGAGAAATTAACTGAATGCCAAGGG
hsa_circ_0003036_R	TGATTTCGTATGATCCGGTCGC
hsa_circ_0034296_F	TTCCAGCCAGCCTGCTAAAC
hsa_circ_0034296_R	AGCTGTTTCCTCCATTGCTGT
hsa_circ_0000780_F	TTAGCACACTGGTGGAAGATCA
hsa_circ_0000780_R	CCAAGATACAACCAAAAGCCAT
hsa_circ_0003352_F	ACTGTTGGATGCAGATGGATGAG
hsa_circ_0003352_R	GAAATAGCCCAGGGGAAGGAGAT
hsa_circ_0001571_F	CGTGCTGAAAGCCGAGGG
hsa_circ_0001571_R	CCAAAGCCTCCGCTGTCC
hsa_circ_0005791_F	AGAACAGTGAACAAGGTGG
hsa_circ_0005791_R	AAACAGTTAATATCATCCC
hsa_circ_0071106_F	GGTTTTGTTTTGACATAGAAGCTGCTG
hsa_circ_0071106_R	CTGCCTCTTGTAAATGTGAGTCTTTCT

**Table 2:** RNA pull-down probe sequence.

	Probe Name	Probe Sequence
full-length sequence	sense	GTACGAGTGGCTGCTTTACAGAATCTGGTGAAGATAAT GTCCTTATATTATCAGTACATGGAGACATATATGGGTCC TGCTCTTTTTGCAATCACAATCGAAGCAATGAAAAGTGA CATTGATGAGGTGGCTTTACAAGGGATAGAATTCTGGTC CAATGTCTGTGATGAGGAAATGGATTTGGCCATTGAAGC TTCAGAGGCAGCAGAACAAGGACGGCCCCCTGAGCACAC CAGCAAGTTTTATGCGAAGGGAGCACTACAGTATCTGGT TCCAATCCTCACACAGACACTAACTAAACAGGACGAAAA TGATGATGACGATGACTGGAACCCCTGCAAAGCAGCAGG GGTGTGCCTCATGCTTCTGGCCACCTGCTGTGAAGATGA CATTGTCCCACATGTCCTCCCCTTCATTAAAGAACACAT CAAGAACCCAGATTGGCGGTACCGGGATGCAGCAGTGAT GGCTTTTGGTTGTATCTTGGAAGGACCAGAGCCCAGTCA GCTCAAACCACTAGTTATACAG
	anti-sense	CTGTATAACTAGTGGTTTGAGCTGACTGGGCTCTGGTC CTTCCAAGATACAACCAAAAGCCATCACTGCTGCATCC CGGTACCGCCAATCTGGGTTCTTGATGTGTTCTTTAAT GAAGGGGAGGACATGTGGGACAATGTCATCTTCACAGC AGGTGGCCAGAAGCATGAGGCACACCCCTGCTGCTTTG CAGGGGTTCCAGTCATCGTCATCATCATTTTCGTCCTG TTTAGTTAGTGTCTGTGTGAGGATTGGAACCAGATACT GTAGTGCTCCCTTCGCATAAAACTTGCTGGTGTGCTCA GGGGGCCGTCCTTGTTCTGCTGCCTCTGAAGCTTCAAT GGCCAAATCCATTTCCTCATCACAGACATTGGACCAGA ATTCTATCCCTTGTAAAGCCACCTCATCAATGTCACTTT TCATTGCTTCGATTGTGATTGCAAAAAGAGCAGGACCC ATATATGTCTCCATGTACTGATAATATAAGGACATTAT CTTCACCAGATTCTGTAAAGCAGCCACTCGTAC
joint sequence	sense	CCACTAGTTATACAGGTACGAGTGGCTGCT
	anti-sense	AGCAGCCACTCGTACCTGTATAACTAGTGG

## Data Availability

Data are contained within the article.
